# The general PTS component HPr determines the preference for glucose over mannitol

**DOI:** 10.1038/srep43431

**Published:** 2017-02-22

**Authors:** Mangyu Choe, Young-Ha Park, Chang-Ro Lee, Yeon-Ran Kim, Yeong-Jae Seok

**Affiliations:** 1 School of Biological Sciences and Institute of Microbiology, Seoul National University, Seoul 00826, Korea; 2 Department of Biological Sciences, Myongji University, Yongin, Gyeonggido 17058, Republic of Korea

## Abstract

Preferential sugar utilization is a widespread phenomenon in biological systems. Glucose is usually the most preferred carbon source in various organisms, especially in bacteria where it is taken up via the phosphoenolpyruvate:sugar phosphotransferase system (PTS). The currently proposed model for glucose preference over non-PTS sugars in enteric bacteria including *E. coli* is strictly dependent on the phosphorylation state of the glucose-specific PTS component, enzyme IIA^Glc^ (EIIA^Glc^). However, the mechanism of the preference among PTS sugars is largely unknown in Gram-negative bacteria. Here, we show that glucose preference over another PTS sugar, mannitol, is absolutely dependent on the general PTS component HPr, but not on EIIA^Glc^, in *E. coli*. Dephosphorylated HPr accumulates during the transport of glucose and interacts with the mannitol operon regulator, MtlR, to augment its repressor activity. This interaction blocks the inductive effect of mannitol on the mannitol operon expression and results in the inhibition of mannitol utilization.

The preferential utilization of a certain carbon source over others has been observed in various model organisms. Carbon catabolite repression (CCR) is generally regarded as the regulatory mechanism to ensure sequential utilization of carbohydrates in microorganisms^1^. Sugars transported by the phosphoenolpyruvate:sugar phosphotransferase system (PTS) are generally believed to be preferred over non-PTS carbon sources in various bacterial species^2^,^3^.

The PTS catalyzes the concomitant phosphorylation and translocation of many sugars across the cytoplasmic membrane^2^. It is a multicomponent system that consists of two general components, enzyme I (EI) and HPr, required for the uptake of most PTS sugars and several sugar-specific enzyme IIs (EIIs). Each EII complex usually has three domains: two cytosolic domains (EIIA and EIIB) and one membrane domain forming the sugar translocation channel (EIIC)^4^. EI transfers a phosphoryl group from PEP to HPr, and HPr then sequentially transfers the phosphoryl group through the various EIIAs and EIIBs to the incoming sugars. Therefore, the phosphorylation states of the PTS components change depending on the availability of a PTS sugar substrate^5^. The ratio of phosphorylated to dephosphorylated PTS components serves as an input signal for the regulation of various metabolic processes including CCR^3^,^4^.

The molecular mechanism for inhibition of expression and/or activity of proteins required for the transport and metabolism of less-preferred carbon sources in the presence of a preferred carbon source has been most extensively studied in *E. coli*, and the best-studied example of this CCR is the glucose-lactose diauxie, which was first observed by Jacques Monod in 1942^6^. The current model for glucose preference over other sugars involves inducer exclusion and induction prevention^3^, both of which are strictly dependent on the phosphorylation state of EIIA^Glc^ (also called Crr for catabolite repression resistant) in *E. coli* (Supplementary Fig. 1). During glucose consumption, EIIA^Glc^ is dephosphorylated. Because dephospho-EIIA^Glc^ can interact with and inhibit several non-PTS permeases including the lactose permease^7^, transport and/or metabolism of less preferred carbon sources is prevented in the presence of glucose. This phenomenon is called “inducer exclusion.” When glucose is exhausted, however, EIIA^Glc^ is phosphorylated and phospho-EIIA^Glc^ then stimulates adenylyl cyclase, an enzyme converting ATP into cyclic AMP (cAMP)^8^. Because cAMP binds to the cAMP receptor protein CRP and the resulting complex functions as a transcription activator, expression of numerous genes and operons required for the transport and/or metabolism of less preferred carbon sources can be induced. Consequently, the cAMP synthesis required for the induction of these genes and operons is prevented during growth on glucose. This is termed “induction prevention”. Therefore, EIIA^Glc^ has been regarded as the central processing unit of CCR in *E. coli* and other enteric bacteria^2^.

Although the current model for CCR may explain the preferential utilization of PTS sugars over non-PTS carbon sources, preference among PTS sugars cannot be explained by inducer exclusion nor by induction prevention. Like glucose, mannitol has also been perceived as a preferred PTS carbon source over some other PTS sugars such as sorbitol, galactitol and fructose in *E. coli*^6^,^9^. Although no diauxie was observed when cells were provided simultaneously with glucose and mannitol, the activity of the mannitol transport system was shown to be inhibited in the presence of glucose in *E. coli*^10^. Such hierarchical utilizations among PTS sugars have been observed in various Gram-negative and in Gram-positive bacteria as well^7^. While competition among various EIIs for phospho-HPr has been suggested as a possible mechanism of the preference among PTS sugars^7^, this possibility has not been experimentally addressed. Therefore, in this study, the molecular mechanism of the preference between glucose and mannitol in *E. coli* was investigated.

## Results

### **The preferential utilization of glucose over mannitol is independent of EIIA** ^**Glc**^

**Figure 1:**
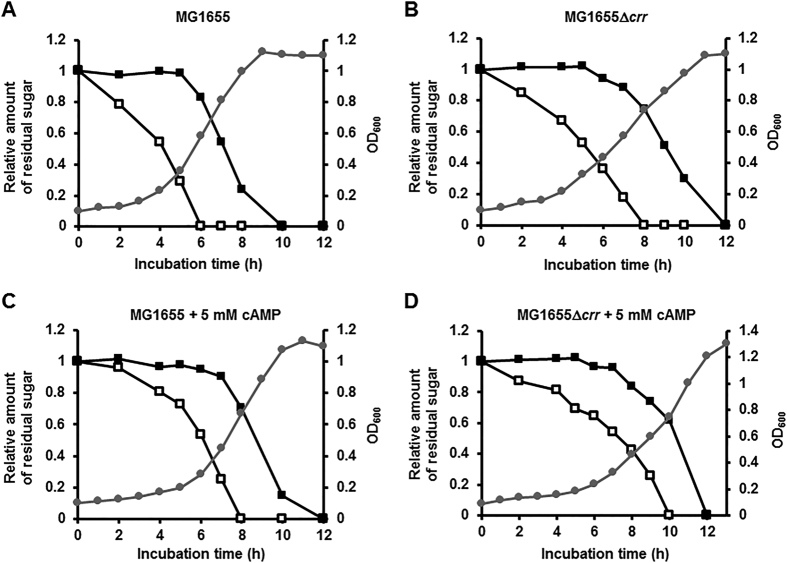
The preferential utilization of glucose over mannitol is independent of the *crr* mutation and cAMP. Wild type (**A**,**C**) and a *crr* deletion mutant (**B**,**D**) of the *E. coli* K12 strain MG1655 were grown in M9 minimal medium supplemented with 0.04% glucose and 0.08% mannitol in the absence (**A**,**B**) and presence (**C**,**D**) of 5 mM cAMP with shaking at 200 rpm. Growth rates (optical density at 600 nm, gray lines with circles) and the concentrations of sugars (open squares for glucose and closed squares for mannitol) remaining in the medium were then measured as a function of incubation time as described in the Materials and Methods section. Representative data from three independent and reproducible measurements are shown.

The currently accepted model of CCR postulates that during growth on glucose dephosphorylated EIIA^Glc^ impedes the transport and/or metabolism of less-favored non-PTS sugars. To verify that EIIA^Glc^ is not implicated in the preference of glucose over the PTS sugar mannitol, we performed a sugar preference test in an *E. coli* mutant deleted of the *crr* gene encoding EIIA^Glc^ (Fig. 1). It is known that glucose can be transported either by the glucose PTS or by the mannose PTS (encoded by *manXYZ*) in *E. coli* and other enteric bacteria, although the mannose PTS can be less efficient than the glucose PTS in uptake and phosphorylation of glucose in *E. coli*^7^,^11^. While the glucose PTS-defective (*crr* and *ptsG*) mutants could still ferment glucose and mannose, mutants with defects in both PTSs were unable to grow on glucose or mannose, implying that glucose can be transported via the mannose PTS when the glucose PTS was blocked^11^,^12^. In agreement with this earlier observation, the *crr* mutant could grow on glucose, although its growth rate was slightly reduced compared to an otherwise isogenic wild-type (WT) MG1655 strain (Fig. 1A,B). As previously reported^10^,^13^, glucose was preferred over mannitol in the WT strain (Fig. 1A). Interestingly, glucose was still preferred over mannitol in the *crr* mutant (Fig. 1B). This observation therefore indicates that the preferential utilization of glucose over mannitol does not involve inducer exclusion by EIIA^Glc^ and also rules out the possibility that the hierarchical utilization among PTS sugars is due to competition between various EIIs for phospho-HPr^7^.

Because the *crr* mutant is known to sustain significantly lower cAMP levels compared to the WT strain^14^,^15^, it may be argued that glucose preference over mannitol in the *crr* mutant could be due to a decreased expression of the *mtl* operon which is known to belong to the CRP regulon^16^. Therefore, we tested the effect of induction prevention on glucose preference over mannitol. Even with the addition of exogenous cAMP to 5 mM, the glucose inhibition of mannitol utilization was still apparent in both WT and *crr* mutant strains (Fig. 1C,D), even though the addition of cAMP slightly decreased the growth rate of the two strains as previously reported^17^. These data suggest that the induction prevention is not responsible for glucose preference over mannitol. It should be noted that previous studies have also demonstrated that the addition of exogenous cAMP did not prevent the preferred utilization of glucose over lactose and melibiose while it abolished the diauxic lag^18^,^19^.

### **Glucose preference over mannitol is accomplished by repression of the** ***mtl*** **operon**

**Figure 2:**
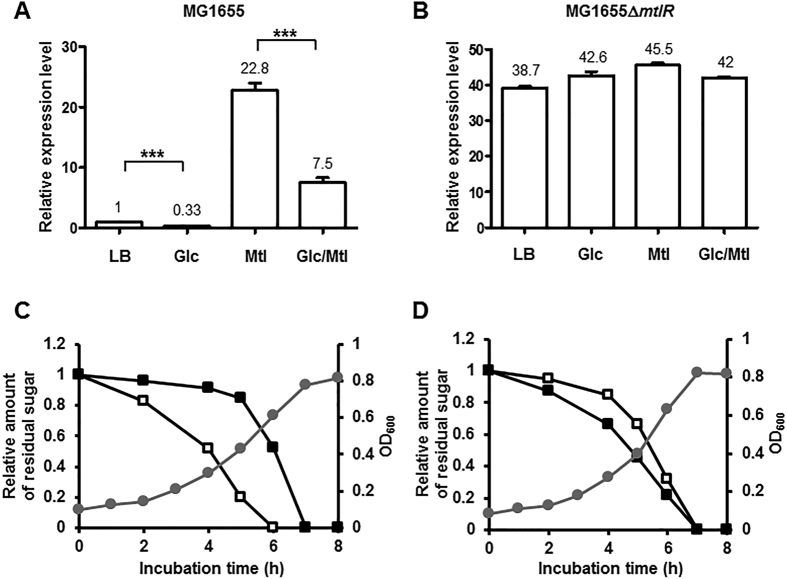
Inactivation of *mtlR* completely abolishes the glucose-dependent repression of the *mtl* operon and glucose preference over mannitol. (**A**,**B**) Total RNA was isolated from wild type (**A**) and an *mtlR* deletion mutant (**B**) of *E. coli* MG1655 grown to early exponential phase in LB medium or LB medium containing the indicated sugars (0.2% each when added alone, or 0.1% each when added in combination), and the expression level of the *mtl* operon was then quantified by qRT-PCR as described in the Materials and Methods section. Representative data (mean ± SD) from three independent experiments (n = 3 each) are shown. Statistical significance was determined by Student’s *t*-test (***P < 0.001). Glc, glucose; Mtl, mannitol; Glc/Mtl, glucose + mannitol. (**C**,**D**) Wild type (**C**) and an *mtlR* deletion mutant (**D**) of *E. coli* MG1655 were grown in M9 minimal medium supplemented with 0.04% glucose and 0.04% mannitol. Growth rates (optical density at 600 nm, gray lines with circles) and the concentrations of sugars (open squares for glucose and closed squares for mannitol) remaining in the medium were then measured as a function of incubation time. Experiments were repeated at least three times with reproducible results.

The *mtl* operon consists of *mtlADR*, where *mltA* encodes the mannitol PTS permease (EIIABC^Mtl^), and *mtlD* encodes mannitol-1-phosphate 5-dehydrogenase converting mannitol-1-phosphate to fructose-6-phosphate in a reversible reaction, and *mtlR* encodes the transcriptional repressor of the *mtl* operon^16^. Although MtlR lacks any known DNA-binding structural motifs and its binding to a specific DNA operator has never been shown, the inactivation of MtlR leads to derepression of the *mtl* operon^16^,^20^. It is generally accepted that the hierarchical utilization of carbon sources is accomplished by repression of the synthesis of the enzymes necessary for the transport and/or metabolism of less favored carbon sources in the presence of a preferred sugar^2^,^3^. For example, in enteric bacteria, the inactivation of LacI relieves the repression of the *lac* operon and eliminates glucose repression^21^. To test whether this is also true or not for glucose preference over mannitol, we constructed a mutant lacking MtlR. As previously shown^10^,^22^, the expression of *mtlA* was derepressed in the presence of mannitol alone, whereas it was repressed in the presence of glucose (Fig. 2A). Moreover, glucose significantly decreased the inductive effect of mannitol on the *mtlA* expression. Therefore, we examined whether the inactivation of MtlR leads to the derepression of the *mtl* operon and thereby influences the preference of glucose over mannitol in a similar manner as the inactivation of LacI abolishes the glucose-lactose diauxie. While glucose dramatically decreased the inductive effect of mannitol on the *mtlA* transcription in a WT strain, this glucose effect was completely abolished in the *mtlR* mutant strain (Fig. 2B). This observation led us to assume that the preferential utilization of glucose over mannitol might be related to an increase in the repressor activity of MtlR in the presence of glucose. To verify this assumption, we first examined the effect of *mtlR* mutation on the sugar preference. As previously reported^10^,^13^, glucose was preferred over mannitol in the WT strain (Fig. 2C). However, the inactivation of MtlR completely abolished glucose preference over mannitol (Fig. 2D), implying that the glucose inhibition of mannitol utilization is accomplished by enhancing repression of the *mtl* operon by MtlR in the presence of glucose.

To test the effect of the expression level of genes required for the transport and metabolism of mannitol on the hierarchical utilization of PTS sugars, sugar preference was tested with WT strains harboring a pBR322-derived expression vector for MtlA, MtlD, or both under the control of the constitutive *cat* promoter of pACYC184. As expected, the preferential utilization of glucose over mannitol was observed in a WT strain harboring pBR322 (Supplementary Fig. 2A). However, similar to the mutant lacking *mtlR* (Fig. 2D), glucose preference was completely abolished in the strain constitutively expressing MtlA alone (Supplementary Fig. 2B). The constitutive expression of MtlD alone resulted in moderate growth retardation but minimally affected glucose preference (Supplementary Fig. 2C). The strain expressing both MtlA and MtlD also displayed retarded growth, but the sugar preference was completely reversed in this strain (Supplementary Fig. 2D). Although we are not able to explain the reason for the growth retardation by constitutive MtlD expression at present, our results support that the preference of glucose over mannitol in *E. coli* is due to the repression of the *mtl* operon, especially to a decreased expression of *mtlA*, in the presence of glucose.

### Dephosphorylated HPr specifically interacts with MtlR

Glucose repression of the *mtl* operon may be accomplished either by regulating MtlR itself, as shown for the regulation of the Mlc activity by the phosphorylation state-dependent interaction with the glucose PTS permease EIICB^Glc^ 23,24, or by regulating the transport (by MtlA) and/or metabolism (by MtlD) of mannitol, as exemplified by the regulation of sugar permeases by the phosphorylation state-dependent interaction with EIIA^Glc^ 19,25. To search for a possible target mediating the glucose-dependent repression of the *mtl* operon, we constructed in-frame deletion mutants of each of the *mtl* operon genes. While the repression of the *mtl* operon by glucose was abolished in *mtlR* and *mtlA* deletion strains, it was still maintained in the *mtlD* mutant strain (Supplementary Fig. 3). Therefore, we excluded the possibility of MtlD as the target mediating the glucose repression.

**Figure 3:**
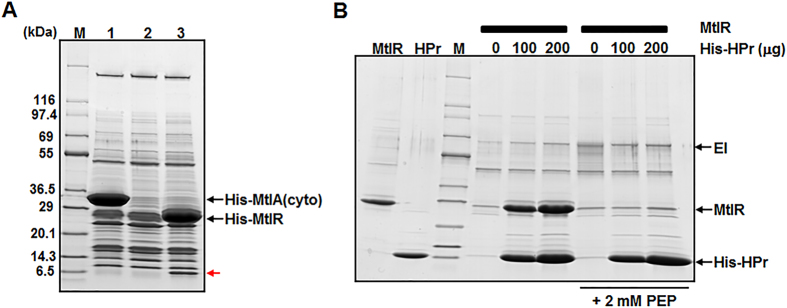
Specific interaction between dephosphorylated HPr and MtlR. (**A**) Protein ligand fishing experiment was performed to search for protein(s) interacting with MtlR and the cytosolic region (residues 348–637) covering the EIIA and EIIB domains of MtlA. The cell lysate prepared from *E. coli* MG1655 grown in 500 ml of LB was divided into three aliquots and mixed with buffer A (lane 2), 500 μg of the purified cytosolic region of MtlA with a His tag at the N-terminus (His-MtlA(cyto), lane 1), and 400 μg of purified His-MtlR (lane 3), respectively. Each mixture was subjected to TALON metal affinity chromatography and proteins bound to the column were analyzed by SDS-PAGE using a 4–20% gradient gel (KOMABIOTECH) and stained with Coomassie brilliant blue R-250. Ezway^TM^ Protein Blue MW Marker (KOMABIOTECH) was used as the molecular mass markers (lane M). The protein band bound specifically to His-MtlR is indicated by a red arrow. (**B**) Untagged MtlR (200 μg) was mixed with 5 μg of EI, 50 μl of TALON metal affinity resin, and various amounts of His-HPr (0, 100, 200 μg) in the absence or presence of 2 mM PEP as indicated. These mixtures were then subjected to pull-down assays to examine the specificity of the interaction between MtlR and HPr and its dependence on the phosphorylation state of HPr. After agitation for 10 min at 4 °C, the protein-bound resin was washed with buffer A containing 10 mM imidazole and eluted with 50 μl of 2X SDS loading buffer. The eluted protein samples (20 μl each), along with MtlR and His-HPr proteins, were analyzed by 4–20% SDS-PAGE, followed by staining with Coomassie brilliant blue R-250. Lane M indicates Ezway^TM^ Protein Blue MW Marker.

To further pinpoint the specific target mediating the glucose effect, protein ligand fishing experiments were performed using the N-terminally His_6_-tagged MtlR and the cytoplasmic domain of MtlA^26^ as bait, as previously described^27^,^28^,^29^,^30^,^31^,^32^. Crude lysate prepared from *E. coli* MG1655 cells was mixed with TALON metal affinity resin (Clontech Laboratories, Inc.) in the presence or absence of His-tagged bait and then subjected to pull-down assays. After brief washes, proteins bound to the resin were eluted with 150 mM imidazole. Analysis of the eluted proteins by SDS-PAGE followed by staining with Coomassie brilliant blue revealed that a protein migrating near the 6.5-kDa protein standard was observed only in the fraction containing His-MtlR as bait (lane 3 in Fig. 3A). Interestingly, we could not detect any protein eluted exclusively in the MtlA-containing fraction (lane 1 in Fig. 3A). Peptide mass fingerprinting by MALDI-TOF MS analysis after in-gel tryptic digestion identified the protein specifically eluted with MtlR as the histidine-containing phosphocarrier protein HPr of the PTS. PTS-mediated regulatory functions are usually based on the phosphorylation state-dependent interactions with their target proteins^4^,^28^,^32^. To confirm the specific interaction between HPr and MtlR and to test whether this interaction could be affected by the phosphorylation state of HPr, we purified untagged MtlR and His-HPr. Because it is well-established that HPr can be phosphorylated *in vitro* by incubation with EI and PEP^32^, MtlR was mixed with EI and increasing amounts of His-HPr in the absence or presence of PEP and subjected to TALON metal affinity chromatography. As shown in Fig. 3B, a concentration-dependent interaction of MtlR was observed in the absence, but not presence of PEP, indicating that only the dephosphorylated form of HPr can interact with MtlR.

To determine the dissociation constant (K_D_) for the binding of MtlR to HPr, HPr was immobilized on a CM5 sensor chip and various concentrations of MtlR were applied to the HPr surface to conduct surface plasmon resonance (SPR) spectroscopy with a BIAcore 3000 system (GE Healthcare Life Sciences)^28^. The SPR signal increased as a function of MtlR concentration (Supplementary Fig. 4). The K_D_ for the MtlR-HPr interaction was determined using BIAevaluation software to be approximately 6.22 × 10^−8^ M, assuming a 1:1 interaction.

To confirm the tight interaction between HPr and MtlR and to determine the binding stoichiometry, we compared the elution profile from a gel filtration column (Superose 12 10/300 GL; GE Healthcare Life Sciences) of the complex with the profiles of individual proteins (Supplementary Fig. 5). MtlR alone was eluted as a symmetric peak at approximately 11.6 ml, corresponding to the tetrameric form (monomer = approximately 22.0 kDa), whereas HPr (approximately 9.1 kDa) was eluted at approximately 14.5 ml. When the mixture of MtlR and HPr was subjected to gel filtration chromatography, the peak at approximately 11.6 ml was shifted to approximately 11.3 ml, with a concomitant slight decrease in the peak at approximately 14.5 ml (Supplementary Fig. 5A). When the gel filtration fractions were analyzed by SDS-PAGE, those eluted at approximately 11.3 ml were resolved into two bands migrating at the positions expected for HPr and MtlR (Supplementary Fig. 5B). Based on the band intensities of the two proteins in these fractions, it appears that HPr interacts with MtlR in a 1:1 ratio and forms a heterooctamer. The low K_D_ value obtained using the BIAcore 3000 system explains that the MltR-HPr complex was tight enough to survive during gel filtration chromatography. Taken together, our data show that only the dephosphorylated form of HPr specifically interacts with MtlR in a 1:1 ratio to form a heterooctamer.

### **Dephosphorylated HPr inhibits derepression of the** ***mtl*** **operon by mannitol**

**Figure 4:**
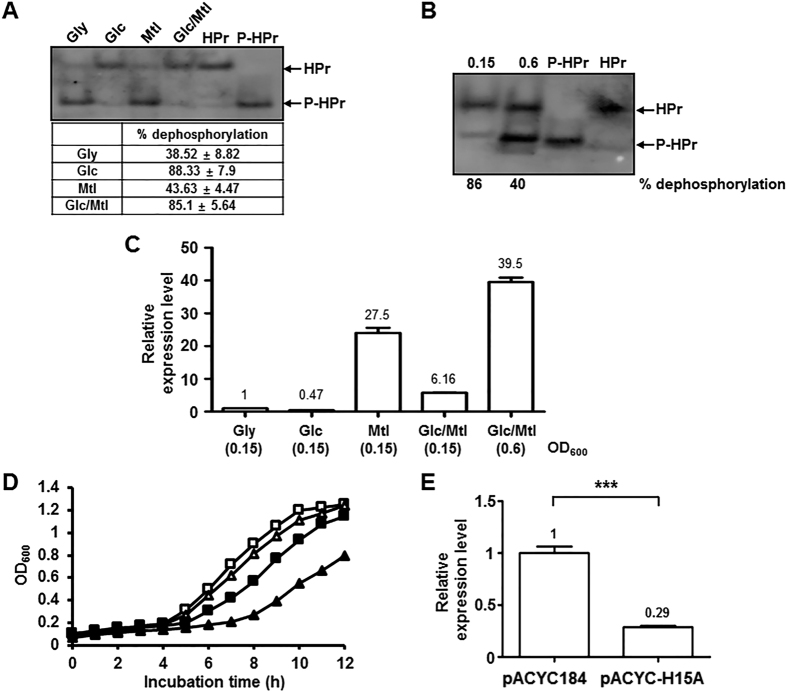
Dephosphorylated HPr inhibits derepression of the *mtl* operon by mannitol. (**A**) *E. coli* MG1655 was grown in M9 minimal medium containing the indicated sugars (0.04% each). The phosphorylation state of HPr was determined when OD_600_ reached 0.15 as described in the Materials and Methods section. Purified HPr (2 ng) was used as a positive control. Band intensities were analyzed using Multi Gauge version 3.0 software. The table below the gel shows the percentage of dephospho-HPr over total HPr with means and standard deviations from three independent experiments. (**B**) The phosphorylation state of HPr was determined in *E. coli* MG1655 cells grown in M9 minimal medium containing 0.04% glucose and 0.04% mannitol at OD_600_ 0.15 and 0.6. Purified HPr (2 ng) was used as a positive control. (**C**) The wild-type *E. coli* MG1655 was cultivated as in (**A**), and harvested at indicated OD_600_. The expression level of *mtlA* was then quantified by qRT-PCR. Representative data (mean ± SD) from three independent experiments (n = 3 each) are shown relative to that observed in glycerol (Gly)-grown cells, and statistical significance was determined by Student’s *t*-test (***P < 0.001). (**D**) Growth of the MG1655 strain harboring the control vector pACYC184 (square symbols) or pACYC-H15A expressing an unphosphorylatable form of HPr (triangle symbols) was monitored in M9 minimal medium supplemented with 0.2% glucose (open symbols) or mannitol (filled symbols) as the sole carbon source at 37 °C. (**E**) The wild-type K12 strain MG1655 harboring pACYC184 or pACYC-H15A was cultivated in M9 minimal medium containing 0.2% mannitol, harvested at early exponential phase, and the transcript level of *mtlA* was measured by qRT-PCR. Representative data (mean ± SD) from three independent experiments (n = 3 each) are shown, and statistical significance was determined by Student’s *t*-test (***P < 0.001).

We have previously shown that HPr was predominantly in the phosphorylated state in LB medium and LB medium supplemented with a non-PTS carbon source, glycerol, whereas it was almost completely dephosphorylated in the presence of glucose^32^. Since HPr is a general PTS component commonly used for many PTS sugars, we first examined the phosphorylation state of HPr in the presence of various PTS sugars in M9 minimal medium (Fig. 4A). It should be noted that because the histidine residue phosphorylated at the N1 position in HPr is extremely unstable at pH < 9.0^32^,^34^, the exposure of samples to pH < 9.0 was minimized after the phosphorylation state of HPr was fixed and cells were lysed in culture medium without any cell harvesting step by adding NaOH^32^. Even though HPr is shared among several PTS sugars, its phosphorylation state significantly varied depending on the type of PTS carbohydrate. To measure the effect of glucose on the phosphorylation state of HPr and the expression of the *mtl* operon, cells were cultivated in M9 minimal medium containing 0.04% glucose, 0.04% mannitol or both sugars. While HPr existed mostly in the dephosphorylated form in the presence of glucose, approximately 61 and 56% of HPr proteins were phosphorylated in the presence of glycerol and mannitol, respectively (Fig. 4A). When cells were incubated in M9 minimal medium containing both glucose and mannitol (0.04% each), the phosphorylation level of HPr during the glucose-consuming period (OD_600_ ~ 0.15) was similar to that of cells grown in the presence of glucose alone (Fig. 4A,B). When glucose was depleted in M9 medium supplemented with both glucose and mannitol (OD_600_ ~ 0.6), however, HPr phosphorylation increased to a level similar to that of cells grown in the presence of mannitol alone (Fig. 4A,B). Then, to measure the effect of glucose depletion on the expression of the *mtl* operon, the transcriptional levels of *mtlA* were determined in cells grown in M9 medium containing both glucose and mannitol at two different growth phases by qRT-PCR (Fig. 4C). At the early growth phase (OD_600_ ~ 0.15), sugar dependence of *mtlA* expression in M9-based medium was similar to that observed in LB-based medium shown in Fig. 2A. After glucose was depleted (OD_600_ ~ 0.6), however, the expression of the *mtl* operon was completely induced. Since only dephosphorylated HPr could interact with MtlR and the level of dephosphorylated HPr was significantly higher in the presence of glucose than in the presence of mannitol, we assumed that the glucose repression of the *mtl* operon may be accomplished via the augmentation of MtlR activity by dephosphorylated HPr. To verify this assumption, we constructed pACYC-H15A, a pACYC184-derived expression vector for the unphosphorylatable form of HPr (HPr(H15A)) driven by the *E. coli ptsH* promoter, and compared the growth rate and the *mtl* operon expression level between the WT MG1655 strains harboring pACYC184 or pACYC-H15A (Fig. 4D,E). We used the WT MG1655 strain carrying pACYC-H15A in an effort to keep the perturbation of the cellular level of WT HPr minimal and only increase the cellular level of unphosphorylated HPr, which would allow for a simpler interpretation of our experimental findings. The WT strain harboring pACYC-H15A showed a similar growth rate with the WT strain carrying the control vector in medium containing glucose as the sole carbon source (compare curves marked with open symbols in Fig. 4D), indicating that the phosphotransferase activity of WT HPr was not significantly affected by an increase in the expression level of the mutant HPr. However, the expression of HPr(H15A) significantly retarded the growth rate of the MG1655 strain in the medium supplemented with mannitol as the sole carbon source (compare curves marked with filled symbols in Fig. 4D). As shown in Fig. 4E, in the WT strain transformed with pACYC-H15A, the expression of *mtlA* significantly decreased in the presence of mannitol. Therefore, these data suggest that glucose preference over mannitol is accomplished, at least in part, by augmentation of the transcriptional repressor activity of MtlR by dephosphorylated HPr in the presence of glucose.

### The interaction between HPr and MtlR determines glucose preference over mannitol

**Figure 5:**
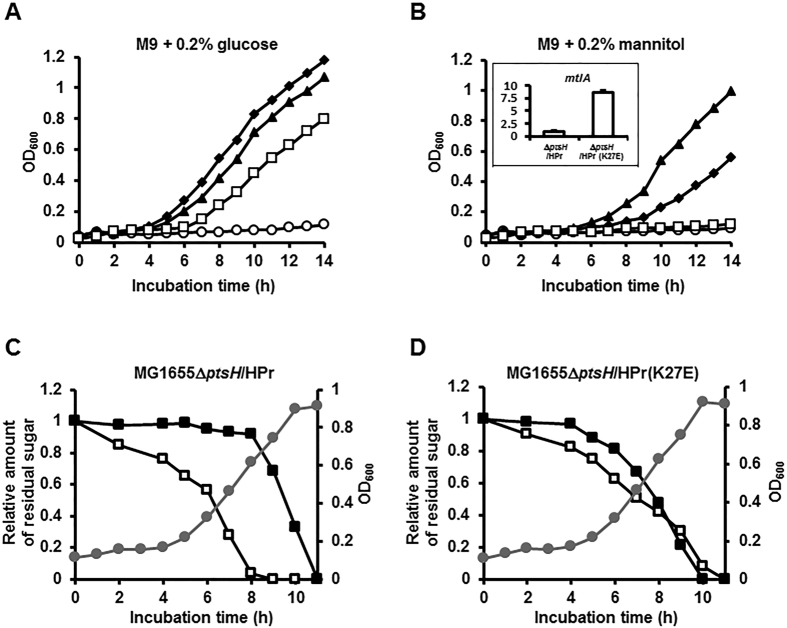
The interaction between HPr and MtlR is sufficient to confer glucose preference over mannitol. (**A**,**B**) Growth of a *ptsH* deletion mutant harboring a pACYC184-derived expression vector for wild-type HPr (pACYC-HPr, closed diamonds), pACYC-HPr(K27E) (closed triangles) or pACYC-HPr(L47A/F48A) (open squares) in M9 medium containing 0.2% glucose (**A**) or 0.2% mannitol (**B**). Growth of the *ptsH* deletion mutant (open circles) is shown as a negative control. The inset in panel (**B**) shows the expression level of *mtlA* measured by qRT-PCR in the *ptsH* deletion mutant harboring pACYC-HPr(K27E) relative to that in the strain harboring pACYC-HPr. (**C**,**D**) The *ptsH* deletion strain harboring pACYC-HPr (**C**) or pACYC-HPr(K27E) (**D**) was grown in M9 minimal medium supplemented with 0.04% glucose and 0.04% mannitol. Growth rates (optical density at 600 nm, gray lines with circles) and the concentrations of sugars (open squares for glucose and closed squares for mannitol) remaining in the medium were measured as a function of incubation time. Representative data from three independent and reproducible measurements are shown.

We anticipated that if the interaction between dephosphorylated HPr and MtlR is sufficient to confer the preference of glucose over mannitol, a mutant HPr, which still retains the phosphotransferase activity required to transport both glucose and mannitol but is no longer able to interact with MtlR, may abolish the preference between glucose and mannitol. Therefore, we purified several mutant HPrs from our collection and examined their interaction with MtlR by TALON metal affinity chromatography using His-tagged MtlR as bait. As shown in Supplementary Fig. 6, the interaction with MtlR was almost completely abolished or significantly decreased for several mutant forms of HPr (R17A, K27E, S46D, and L47A/F48A), while the other mutants still retained their interactions with MtlR to a comparable level as the WT HPr. The Arg17 residue near the His15 phosphorylation site is highly conserved, and a previous study has shown that all mutations at Arg17 resulted in the impairment of the phosphocarrier function of HPr with enzyme I^34^. The Ser46 residue is also widely conserved in Gram-positive and Gram-negative bacteria and mutation of Ser46 to Asp was previously shown to decrease the phosphoacceptor activity of HPr from enzyme I by approximately 2000-fold^35^. Therefore, we examined whether the phosphotransferase activity was impaired or not in K27E and L47A/F48A mutants of HPr by determining the growth rate of a *ptsH* deletion mutant harboring a pACYC184-derived expression vector pACYC-HPr, pACYC-HPr(K27E), or pACYC-HPr(L47A/F48A) in M9 medium containing 0.2% glucose or mannitol (Fig. 5A,B). As previously reported^7^, the *ptsH* mutant did not grow in M9 medium supplemented with glucose or mannitol. The expression of the L47A/F48A mutant HPr in trans could rescue the growth defect of the *ptsH* deletion strain in M9 medium supplemented with glucose, but not in mannitol-containing medium. Because the expression of the L47A/F48A mutant HPr in a *ptsH mtlR* double deletion mutant could partially rescue the growth defect on mannitol (Supplementary Fig. 7), it is most likely that the expression level of MtlA in the *mtlR*^+^strain is not sufficient to support growth on mannitol with the MtlA phosphotransferase activity of HPr(L47A/F48A). The *ptsH* mutant strain harboring pACYC-HPr(K27E) showed a similar growth rate to that of the mutant strain harboring pACYC-HPr in the glucose-containing medium. Surprisingly, however, the *ptsH* mutant strain harboring pACYC-HPr showed a significantly slower growth rate than the strain harboring pACYC-HPr(K27E) in M9 minimal medium containing mannitol even though the expression levels of the two HPr proteins were quite similar. It was shown that an increase in dephosphorylated HPr resulted in a decreased expression of the *mtl* operon (Fig. 4C,E) and approximately 44% of HPr existed in the dephosphorylated state in the presence of mannitol (Fig. 4A). Therefore, we assumed that the slower growth rate of the strain expressing WT HPr from the multi-copy plasmid pACYC184 compared to the HPr(K27E)-overproducing strain in the mannitol-containing medium might be due to the augmentation of the MtlR repressor activity by WT HPr dephosphorylated during the transport of mannitol but not by HPr(K27E), which cannot interact with MtlR. Indeed, a significantly greater repression of the *mtlA* expression was observed in the WT HPr-overproducing strain than the HPr(K27E)-overproducing strain even in the presence of mannitol (inset in Fig. 5B). A slower growth of the *ptsH mtlR* double mutant carrying pACYC-HPr(K27E) compared to that of the double mutant carrying pACYC-HPr (Supplementary Fig. 7) clearly shows that HPr(K27E) does not have a higher MtlA phosphotransferase activity than WT HPr. Therefore, we concluded that the faster growth of the *ptsH* mutant carrying pACYC-HPr(K27E) compared to that of the *ptsH* mutant carrying pACYC-HPr (Fig. 5B) is solely due to the lack of its interaction with MtlR. Then, to determine the contribution of the tight interaction between dephosphorylated HPr and MtlR to the preference between glucose and mannitol, the sugar preference experiments were performed with HPr- and HPr(K27E)-overproducing strains. Interestingly, while glucose was still preferred over mannitol in the strain harboring pACYC-HPr (Fig. 5C), glucose preference was almost completely abolished in the strain harboring pACYC-HPr(K27E) (Fig. 5D), and this strain consumed glucose and mannitol simultaneously as did the *mtlR* deletion mutant (Fig. 2D). These data indicate that the interaction between dephosphorylated HPr and MtlR is sufficient to confer the preference of glucose over mannitol. Taken together, the data in this study show that a higher level of the dephosphorylated form of the general PTS component HPr accumulates in the presence of a more favorable sugar, and dephosphorylated HPr determines the preference between the PTS sugars glucose and mannitol by inhibiting the induction of genes required for the transport and metabolism of the less preferred sugar mannitol via direct interaction with the mannitol operon repressor MtlR.

## Discussion

In this study, we demonstrated that the preference between the two PTS sugars glucose and mannitol does not involve inducer exclusion and induction prevention by dephosphorylated EIIA^Glc^, but is determined solely by the phosphorylation state-dependent interaction of HPr with the mannitol operon repressor, MtlR, in *E. coli*. Dephosphorylated HPr, but not its phospho-form, specifically interacted with MtlR (Fig. 3B), and the level of dephosphorylated HPr was significantly higher in the presence of glucose than in the presence of mannitol (Fig. 4A). In the presence of mannitol alone, the expression of the *mtl* operon was remarkably induced (Figs 2A and 4C). However, dephosphorylated HPr inhibited the induction of the *mtl* operon genes and thus the utilization of mannitol by binding to MtlR and enhancing its repressor activity even in the presence of mannitol (Figs 4 and 5). Therefore, a complete loss of glucose preference over mannitol could be caused by *mtlR* deletion (Fig. 2D), additional expression of *mtlA* from a multi-copy plasmid (Supplementary Fig. 2B), or substitution of HPr with HPr(K27E), which still retains the phosphotransferase activity but is unable to interact with MtlR (Fig. 5D), all of which would lead to an increased expression level of the *mtl* operon even in the presence of glucose. Taken together, all observations suggest that the preference of glucose over mannitol is entirely dependent on the prevention of the induction of the mannitol operon genes by dephosphorylated HPr.

Despite considerable efforts, the DNA-binding activity of MtlR has not yet been shown and no DNA binding domain has been found in its crystal structure^16^,^20^. Although we could not detect the direct DNA-binding activity of purified MtlR and the MtlR-HPr complex, genetic evidence supports the direct or the indirect binding of MtlR to the promoter region of the *mtl* operon. Consistent with a previous study^16^, transformation of *E. coli* MG1655 with the multi-copy plasmid pET-43.1a(+), bearing the promoter region of the *mtl* operon (pET-pro) increased *mtlA* expression by a factor of 13.2, which is equivalent to the copy number of pBR322 derivatives, whereas pET-43.1a(+) itself and the plasmid cloned with a DNA fragment covering the 5′-untranslated region (pET-utr) had no significant effect on the *mtlA* transcription level (Supplementary Fig. 8). However, the inductive effect of the plasmid pET-pro was not observed in the *mtlR* mutant. These data suggest that MtlR specifically binds to the promoter region. Because MtlR itself has no DNA binding activity, we assume the existence of a cognate DNA-binding protein of MtlR, as also suggested in a previous report^20^. As the promoter region of the *mtl* operon has been shown to contain five CRP-binding sites^36^, it was reasonable to speculate that the expression of the *mtl* operon might be regulated by a direct interaction between MtlR and CRP. However, we could not detect interaction between MtlR and CRP. Furthemore, transformation of MG1655 with pET-malK carrying the promoter region of the *malK* operon containing four consecutive CRP-binding sites^37^ had little effect on the *mtlA* transcription level. These data suggest that repression of the *mtl* operon by MtlR is not mediated through a direct interaction with CRP. The increase of *mtlA* transcription in the WT MG1655 strain carrying pET-pro by a factor of approximately the copy number of the promoter DNA suggest that MtlR and its cognate DNA-binding protein are present in a very low copy number in the cells, as also suggested by a previous report^16^.

Our data in this study show that expression of the *mtl* operon is induced in the presence of mannitol but HPr significantly enhances the repressor activity of MtlR to antagonize the inductive effect of mannitol in the presence of glucose. In a previous study, mannitol, but not mannitol-1-phosphate, was suggested as an inducer of the *mtl* operon^16^. However, when we determined the inductive effect of mannitol on the *mtl* operon in the *mtlA* or *mtlD* deletion mutant, the significant induction by mannitol was observed in the *mtlD* mutant, but not in the *mtlA* mutant (Supplementary Fig. 3), suggesting that the translocation and/or phosphorylation of mannitol by MtlA is important for the induction of the *mtl* operon. However, we could not detect direct interactions of either mannitol or mannitol-1-phosphate with purified MtlR or the MtlR-HPr complex.

Considering the low K_D_ value of the MtlR-HPr complex and cellular concentrations of MtlR and HPr, a significant fraction of MtlR should exist complexed with HPr even in the absence of glucose. Although the expression of the *mtl* operon became derepressed in the presence of mannitol (Fig. 4C), the data in Fig. 5 suggest that a significant portion of MtlR exists complexed with HPr in the strain expressing wild-type HPr even during growth on mannitol. It should be noted that, in the presence of both glucose and mannitol, the expression level of *mtlA* is not fully induced but ~4.5 fold lower compared with the level in cells grown on mannitol alone, whereas significantly increased (~13 fold) compared with the level in cells grown on glucose alone (Fig. 4C). However, this level of *mtl* operon expression is likely insufficient to meet a minimum threshold required for mannitol utilization, indicating that mannitol consumption is delicately regulated in the presence of glucose. This may reflect the need for the high-affinity interaction between MtlR and HPr. At present, we are unable to explain how the induction signal of mannitol is transduced, but assume that the K_D_ of the MtlR-HPr complex might increase in the presence of other factors such as the cognate DNA-binding protein of MtlR and an inducer (probably mannitol-1-phosphate) of the *mtl* operon.

As a common PTS protein, HPr is highly conserved in Gram-positive and Gram-negative bacteria^38^. HPr has long been recognized as the key player in CCR that regulates the activities of metabolic enzymes and transcriptional regulators in several Gram-positive bacteria^3^,^4^. As would be anticipated from the high similarity, increasing numbers of regulatory functions are being discovered for HPr in Gram-negative bacteria. HPr was first shown to regulate glycogen phosphorylase by direct interaction^39^, and then activate the antiterminator BglG by phosphorylation^40^ in *E. coli*. Recently, it was also shown that only unphosphorylated *E. coli* HPr formed a tight complex with Rsd and thus antagonized its anti-σ^70^ activity^32^,^41^. It is interesting to note that amino acid residues R17, K27, S46, L47, and/or F48 of HPr are involved in the phosphorylation-dependent interaction with both Rsd and MtlR in *E. coli*^41^ (Supplementary Fig. 6). All of these residues are clustered on the surface of HPr and may define an interaction patch. Therefore, these findings suggest the possibility that these residues might be commonly used for regulation of its target proteins by the phosphorylation-dependent protein-protein interactions in *E. coli*.

In this study, we provide a novel mechanism for the CCR, which is independent of EIIA^Glc^ but regulated by the phosphorylation state-dependent interaction with HPr. It would be interesting to show whether the inhibition of the induction of genes for the utilization of a less preferred PTS sugar by dephosphorylated HPr is specific for the preference between glucose and mannitol or widespread for the preference among PTS sugars. As neither HPr nor MtlR possesses a DNA-binding domain, we need to identify a DNA-binding repressor involved in this regulation to fully understand the regulatory mechanism of the *mtl* operon by the HPr-MtlR complex.

## Materials and Methods

### Bacterial strains, plasmids, and culture conditions

The bacterial strains and plasmids used in this study are listed in Supplementary Table 1. Bacterial cells were grown at 37 °C in LB medium or M9 minimal medium containing the indicated amount of sugars in culture flasks with shaking at 200 rpm. All plasmids were constructed using standard polymerase chain reaction (PCR)-based cloning procedures and verified by sequencing. In-frame deletion mutants used in this study were constructed using the pKD46 plasmid as previously described^42^. The *mtlA* and *mtlD* genes were replaced by the chloramphenicol-resistance gene and the *mtlR* gene by the kanamycin-resistance gene.

### Purification of proteins

Proteins with N-terminal His tags used in this study were purified by immobilized metal affinity chromatography (IMAC) using TALON metal affinity resin according to the manufacturer’s instructions (Clontech Laboratories, Inc.). The proteins were eluted with 150 mM imidazole and concentrated using Amicon Ultracel-3K centrifugal filters (Merck Millipore). To remove imidazole and increase purity, the concentrated proteins were chromatographed on a HiLoad 16/60 Superdex 75 prepgrade column (GE Healthcare Life Sciences) equilibrated with buffer A (20 mM HEPES, pH 7.5, 100 mM NaCl, 0.05% β-mercaptoethanol, and 5% glycerol). Untagged proteins were overexpressed and purified using MonoQ^TM^ 10/100 GL and HiLoad 16/60 Superdex 75 prepgrade columns (GE Healthcare Life Sciences) as recently reported^28^. Cells induced to overexpress untagged HPr, EI, EIIA^Glc^, MtlR, or mutated HPr were resuspended in buffer B (20 mM Tris-HCl, pH 8.0, 50 mM NaCl, 0.05% β-mercaptoethanol, and 5% glycerol) and disrupted by two passages through a French pressure cell at 10,000 psi. After centrifugation at 100,000 × *g* for 60 min at 4 °C, the supernatant was applied to a MonoQ^TM^ 10/100 GL column equilibrated with buffer B. Protein elution was carried out by using a 15-column volume gradient of 50–500 mM NaCl in buffer B at a flow rate of 1 ml/min. The fractions containing the desired protein were pooled and concentrated as described above. Concentrated samples were chromatographed on a HiLoad 16/60 Superdex 75 prepgrade column (GE Healthcare Life Sciences) equilibrated with buffer A. The purified proteins were stored at −80 °C until use.

### Ligand fishing experiments using immobilized metal affinity chromatography

*E. coli* MG1655 cells grown overnight at 37 °C in LB medium were harvested and resuspended in buffer A. Cells were disrupted by two passages through a French press cell at 10,000 psi. After centrifugation at 10,000 × *g* for 25 min at 4 °C, the supernatant was divided into aliquots and mixed with either binding buffer as control or a His-tagged protein as bait. Each mixture was incubated with 50 μl of TALON metal affinity resin in 1.5 ml tube at 4 °C for 30 min. After three brief washes with buffer A, the bound proteins were eluted with 50 μl of buffer A containing 150 mM imidazole and analyzed by Coomassie blue staining following SDS-PAGE. Protein bands specifically bound to the His-tagged bait were excised from the gel, and tryptic in-gel digestion and peptide mass fingerprinting were performed as previously described^43^.

### Determination of the amounts of sugars in medium

*E. coli* cells grown overnight in LB medium were harvested, washed once with M9 medium lacking a carbon source and resuspended in the same medium. These cell suspensions were used to inoculate M9 minimal media containing glucose and mannitol to an optical density at 600 nm of 0.1. Cells were cultivated at 37 °C and 1 ml aliquots were withdrawn at various times to determine the residual sugars in culture medium. After centrifugation at 10,000 × *g* for 2 min, sugars remaining in the culture supernatant were analyzed using a Sugar-Pak I column connected to a Dionex Ultimate 3000 High-Performance Liquid Chromatography system equipped with a refractive index detector (Thermo Fisher Scientific). TDW was used as the mobile phase, and the column was maintained at 75 °C during the experiment.

### RNA isolation and qRT-PCR

Cells were cultivated at 37 °C in LB medium or M9 minimal medium supplemented with the indicated amount of sugars and harvested at mid-exponential phase or indicated OD_600_, respectively. Total RNA was prepared using an RNeasy Mini Kit (Qiagen) and genomic DNA was removed using RNase-free DNase I (Promega). From each culture, 2500 ng of total RNA was used to synthesize cDNA using the RNA to cDNA EcoDry^TM^ Premix (Clontech Laboratories, Inc.). cDNAs were diluted 20-fold and subjected to qRT-PCR analyses using gene-specific primers and 2X SYBR Premix Ex Taq II (Takara). Amplification and detection of product were performed using the CFX96 Real-Time System (Bio-Rad). For normalization of the transcript level, *rrsH* was used as a normalization control. The relative expression level was calculated as the difference between the threshold cycle (Ct) of the target gene and the Ct of the reference gene for each template.

### **Determination of the** ***in vivo*** **phosphorylation state of HPr**

The phosphorylation state of HPr was determined as previously described with some modifications^32^. Because 1-phosphohistidine residues are known to be extremely unstable at neutral and acidic pH, exposure of samples to pH < 9.0 was minimized. *E. coli* MG1655 was cultivated in M9 minimal medium containing the indicated sugars. Cell cultures (0.15 ml at indicated OD_600_) were quenched, the phosphorylation states of HPr were fixed, and the cells were disrupted at the same time by mixing with 15 μl of 5 M NaOH, followed by vortexing for 20 s and the addition of 45 μl of 3 M sodium acetate (pH 5.2) and 0.65 ml of ethanol. The samples were centrifuged at 10,000 × *g* at 4 °C for 10 min. The pellet was suspended in 20 μl of native PAGE sample buffer containing 1 M urea, and this sample was immediately analyzed by 16% native PAGE. Protein amounts were normalized to cell density. Western blotting was performed as previously described^32^.

## Additional Information

**How to cite this article:** Choe, M. *et al*. The general PTS component HPr determines the preference for glucose over mannitol. *Sci. Rep.* **7**, 43431; doi: 10.1038/srep43431 (2017).

**Publisher's note:** Springer Nature remains neutral with regard to jurisdictional claims in published maps and institutional affiliations.

## Supplementary Material

Supplementary Information
