# Supplementary material for: The general PTS component HPr determines the preference for glucose over mannitol
Source: Sci Rep. 2017 Feb 22;7:43431. doi: 10.1038/srep43431 (PMC5320558; doi:10.1038/srep43431)
Supplement: Supplementary Information [file srep43431-s1.pdf]

The general PTS component HPr determines the preference for glucose over mannitol

Mangyu Choe[a], Young-Ha Park[a], Chang-Ro Lee[b], Yeon-Ran Kim[a] and Yeong-Jae Seok[a‡]

[a]School of Biological Sciences and Institute of Microbiology, Seoul National University, Seoul 151-742, Korea

[b]Department of Biological Sciences, Myongji University, Yongin, Gyeonggido 449-728, Republic of Korea

[‡]To whom correspondence should be addressed:

Tel: 82-2-880-8827; E-mail: yjseok@snu.ac.kr

Supplementary Informations

Supplementary Table 1

Supplementary Figures 1-8

Supplementary References

Supplementary Table 1. ***Escherichia coli*** **strains and plasmids used in this study.**

Strains or plasmid	Genotype or phenotype	Source or Reference
**Strains**		
MG1655	Wild-type *E. coli* K-12	[1]
MG1655Δ*ptsH*	MG1655 *ptsH*::Km^r	[2]
MG1655Δ*crr*	MG1655 *crr*::Tet^r	Lab stock
MG1655Δ*mtlR*	MG1655 *mtlR*::Km^r	This study
MG1655Δ*mtlA*	MG1655 *mtlA*::Cat^r	This study
MG1655Δ*mtlD*	MG1655 *mtlD*::Cat^r	This study
MG1655Δ*ptsH*Δ*mtlR*	MG1655 *ptsH*::Tet^r, *mtlR*::Km^r	This study
GI698	F$^-$ λ$^-$ *lacI^q lacPL8 ampC*::P$_{trp}$ cI	[3]
GI698Δ*pts*	GI698 *ptsHIcrr*::Km^r	[4]
ER2566	F- λ- f*huA2* [*lon*] *ompT lacZ::T7 gene 1 gal sulA11* Δ(*mcrC-mrr*)*114::IS10 R*(*mcr-73::miniTn10-TetS*)*2 R*(*zgb-210::Tn10*)(*TetS*) *endA1* [*dcm*]	New England Biolabs
**Plasmid**		
pRE1	Expression vector under control of λP$_L$ promoter, Amp^r	[5]
pRE-HIcrr	pRE1-based expression vector for HPr, EI and EIIA^{Glc}	Lab stock
pRE-H(K27E)Icrr	Lys27 of HPr mutated to Glu in pRE-HIcrr	Lab stock
pRE-H(D69E)Icrr	Asp69 of HPr mutated to Glu in pRE-HIcrr	Lab stock
pSP100	pRE1-based expression vector for HPr	[6]
pSP100 (R17A)	Arg17 of HPr mutated to Ala in pSP100	[7]
pSP100 (Q51A)	Gln51 of HPr mutated to Ala in pSP100	[7]
pSP100 (L55A)	Leu55 of HPr mutated to Ala in pSP100	[7]
pSP100 (N12A)	Asn12 of HPr mutated to Ala in pSP100	[7]
pSP100 (L47A/F48A)	Leu47 and Phe48 of HPr mutated to Ala in pSP100	[7]
pACYC184	Cloning vector; Cm^r Tet^r	[8]
pACYC-HPr	*E. coli ptsH* ORF and its promoter cloned between SphI and SalI sites of pACYC-184	Lab stock
pACYC-HPr(H15A)	His15 of HPr mutated to Ala in pACYC-HPr	Lab stock
pACYC-HPr(K27E)	Lys27 of HPr mutated to Glu in pACYC-HPr	This study
pACYC-HPr(L47A/F48A)	Leu47 and Phe48 of HPr mutated to Ala in pACYC-HPr	This study
pET43.1a	Cloning vector; Amp^r	Novagen
pET-MtlR	*E. coli mtlR* ORF cloned between NdeI and XhoI sites of pET43.1a	This study
pET-HisMtlR	His$_6$ tag added to the N-terminus of MtlR in pET-MtlR	This study
pET-HisMtlA (cyto)	His$_6$ tag added to the cytosolic domain (AB) of *E. coli* MtlA cloned between NdeI and XhoI sites of pET43.1a	This study
pET-pro	Promoter region of *E. coli mtl* operon cloned between NdeI and XhoI sites of pET43.1a	This study
pET-utr	5'-untranslated region of *E. coli mtlA* cloned between NdeI and XhoI sites of pET43.1a	This study
pET-malK	Promoter region of *E. coli malK* operon cloned between NdeI and XhoI sites of pET43.1a	This study
pBR322	Cloning vector; Amp^r Tet^r	[9]
pBR322-MtlA	*E. coli mtlA* ORF with constitutive *CAT* promoter cloned between BamHI and SalI sites of pBR322	This study
pBR322-MtlD	*E. coli mtlD* ORF with constitutive *CAT* promoter cloned between BamHI and SalI sites of pBR322	This study
pBR322-MtlAD	*E. coli mtlAD* ORF with constitutive *CAT* promoter cloned between BamHI and SalI sites of pBR322	This study
pKD46	Red recombinase expression plasmid under the control of arabinose inducible promoter	[10]

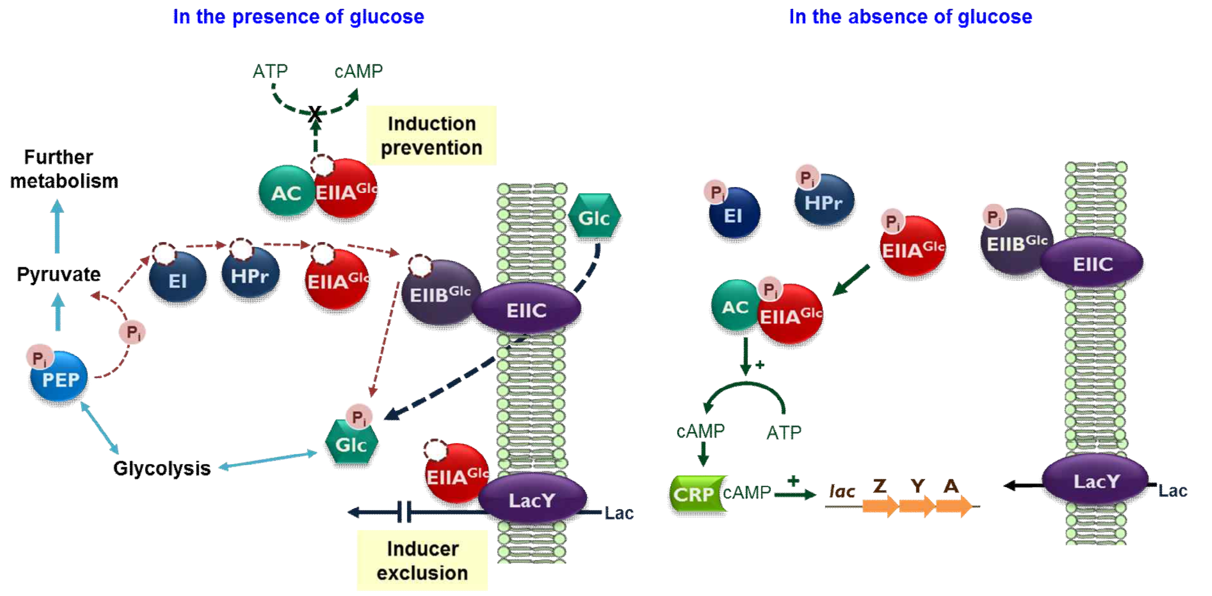

Supplementary Figure 1. A schematic view of the current model for carbon catabolite repression (CCR) in *E. coli*.

Glucose preference over non-PTS carbon compounds is currently explained by inducer exclusion and induction prevention in *E. coli*. In the presence of glucose, the phosphoryl group of phosphoenolpyruvate (PEP) is sequentially transferred through EI, HPr and EII^{Glc} to glucose, and therefore $EIIA^{Glc}$ is predominantly dephosphorylated. Because dephospho-$EIIA^{Glc}$ interacts with and inhibits several non-PTS permeases including the lactose permease (LacY), the transport of less preferred carbon sources is prevented in the presence of glucose. This is called "inducer exclusion." In the absence of glucose, however, $EIIA^{Glc}$ is phosphorylated and stimulates adenylyl cyclase (AC), an enzyme converting ATP into cAMP. CRP complexed with cAMP then activates expression of numerous genes and operons required for metabolism of less preferred carbon sources. Because dephospho-$EIIA^{Glc}$ cannot stimulate AC, the synthesis of cAMP required for the induction of these genes and operon is prevented in the presence of glucose. This is termed "induction prevention." Therefore, the current model for glucose preference over non-PTS sugars is strictly dependent on the phosphorylation state of $EIIA^{Glc}$.

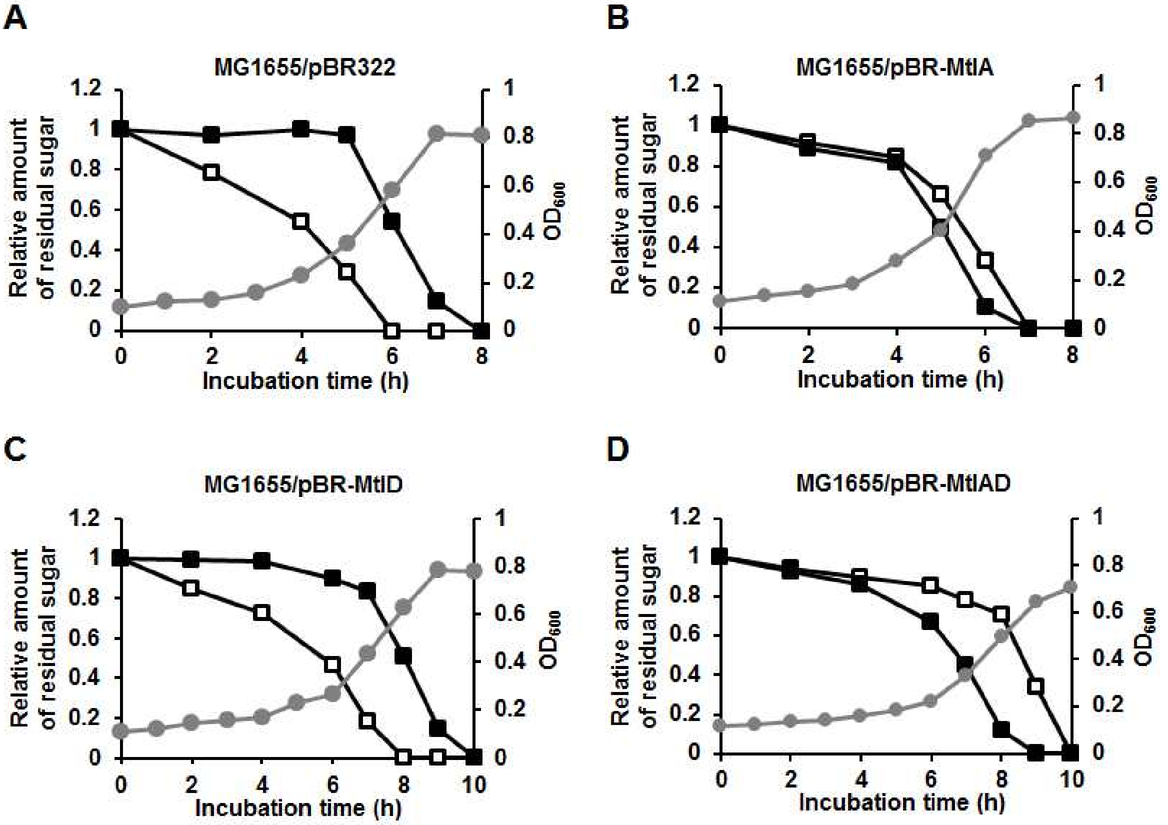

Supplementary Figure 2. Effect of the overexpression of the mannitol operon genes on the sequential utilization of glucose and mannitol.

Wild-type strains harboring the pBR322 control vector (A) or the pBR322-derived expression vector for MtlA (B), MtlD (C) or both (D) were grown in M9 minimal medium supplemented with 0.04% glucose and 0.04% mannitol. Growth rates (optical density at 600 nm, gray lines with circles) and the concentrations of sugars (open squares for glucose and closed squares for mannitol) remaining in the medium were then measured as a function of incubation time. Representative data from three independent experiments are shown here.

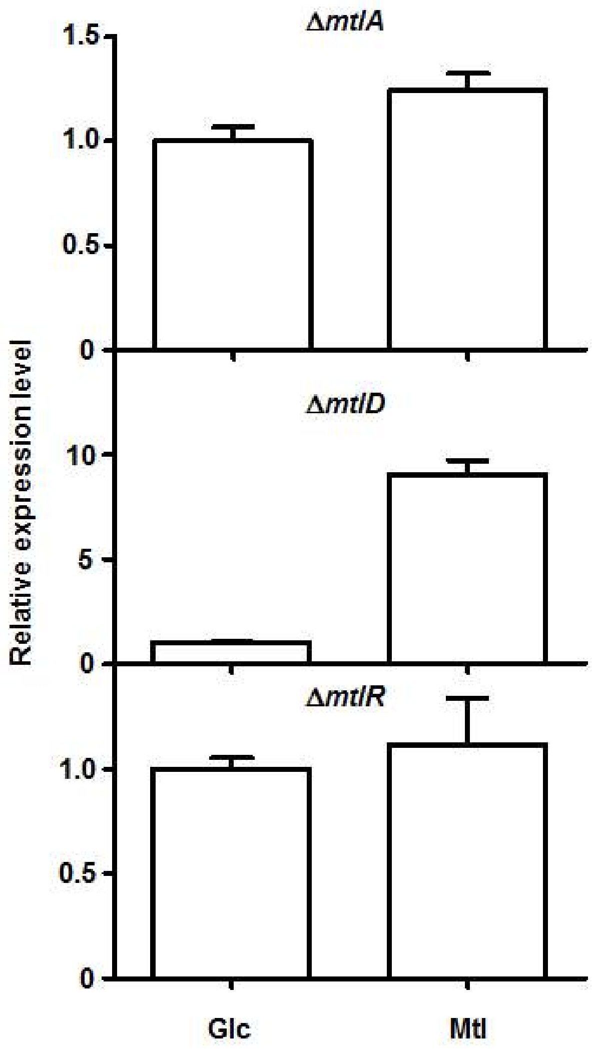

Supplementary Figure 3. Effect of each gene in the *mtl* operon on the glucose-mediated repression of the operon.

Deletion mutants lacking each gene in the *mtl* operon were grown in LB medium containing the indicated sugars (0.2% each), harvested at early exponential phase and the expression level of the *mtl* operon was then quantified by qRT-PCR. While the expression level of *mtlD* was measured in the *mtlA* deletion mutant, the expression level of *mtlA* was measured in *mtlD* and *mtlR* deletion mutants. Representative data (mean ± SD) from three independent experiments (n=3 each) are shown.

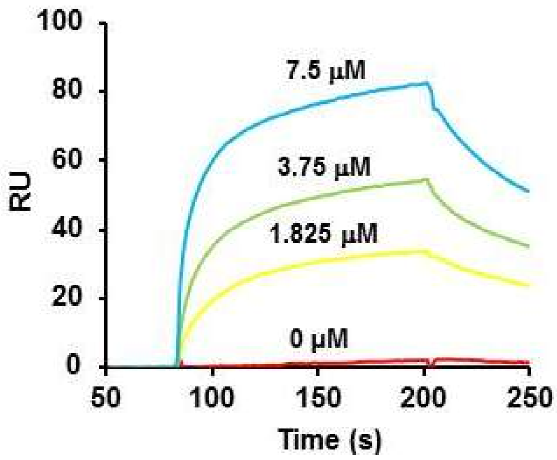

Supplementary Figure 4. Measurement of the dissociation constant (K_D) between HPr and MtlR.

Real-time interaction of MtlR and HPr was monitored by surface plasmon resonance (SPR) detection using a BIAcore 3000 (GE Healthcare Life Sciences) as previously described with some modifications[11]. Purified HPr was immobilized on the carboxymethylated dextran surface of a CM5 sensor chip using a NHS/EDC reaction. The standard running buffer was 20 mM HEPES (pH 7.4) and 100 mM NaCl. All reagents were introduced at a flow rate of 10 μl/min. The indicated concentrations of MtlR were allowed to flow over immobilized HPr. The sensor surface was regenerated between assays by flushing with the standard running buffer at a flow rate of 100 μl/min for 10 min to remove bound analytes. The K_D value between MtlR and HPr was determined using BIAevaluation 2.1 software (GE Healthcare Life Sciences).

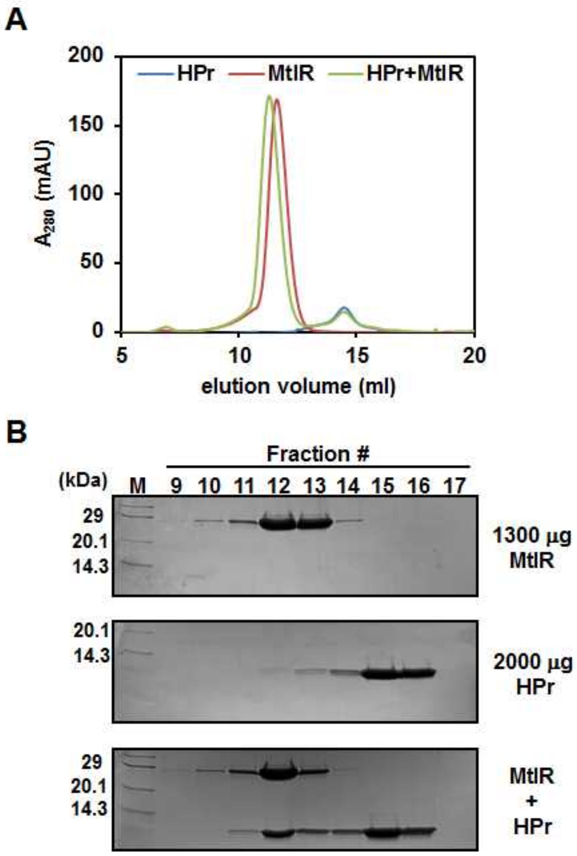

Supplementary Figure 5. Gel filtration chromatography of MtlR, HPr and the MtlR-HPr complex.

Gel filtration chromatography was performed on a Superose 12 10/300 GL column (GE Healthcare Life Sciences) equilibrated with running buffer containing 20 mM HEPES (pH 7.5), 100 mM NaCl, 0.05% β-mercaptoethanol and 5% glycerol at a flow rate of 0.5 ml/min using AKTA FPLC system. (A) Each sample containing 1300 μg of MtlR (red line), 2000 μg of HPr (blue line), or both proteins (green line) in 200 μl of the running buffer was incubated for 10 min on ice and then injected through the column and the three chromatograms recorded at 280 nm were superimposed to compare the elution profiles. (B) Fractions (1 ml) were collected, and 25 μl of each fraction was analyzed by SDS-PAGE followed by staining with Coomassie brilliant blue R-250. Lane M indicates the EZway^{TM} Protein Blue MW Marker (KOMABIOTECH), and the molecular masses (in kDa) of some standards are presented on the left. Numbers indicate fractions from the gel filtration column.

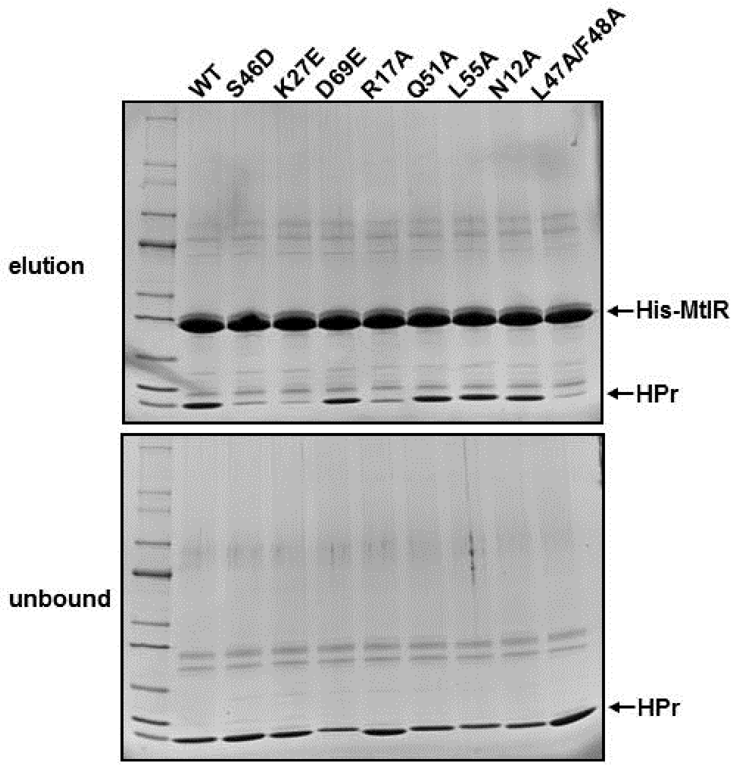

Supplementary Figure 6. Examination of mutant HPrs for their interaction with MtlR. His-MtlR (100 μg) was mixed with mutant HPrs (50 μg each) and then subjected to TALON metal affinity chromatography. After a brief wash with buffer A containing 10 mM imidazole, samples were eluted with 2X SDS loading buffer. Along with unbound samples (lower gel), eluted samples (upper gel) were analyzed by 4-20% SDS-PAGE followed by staining with Coomassie brilliant blue R-250.

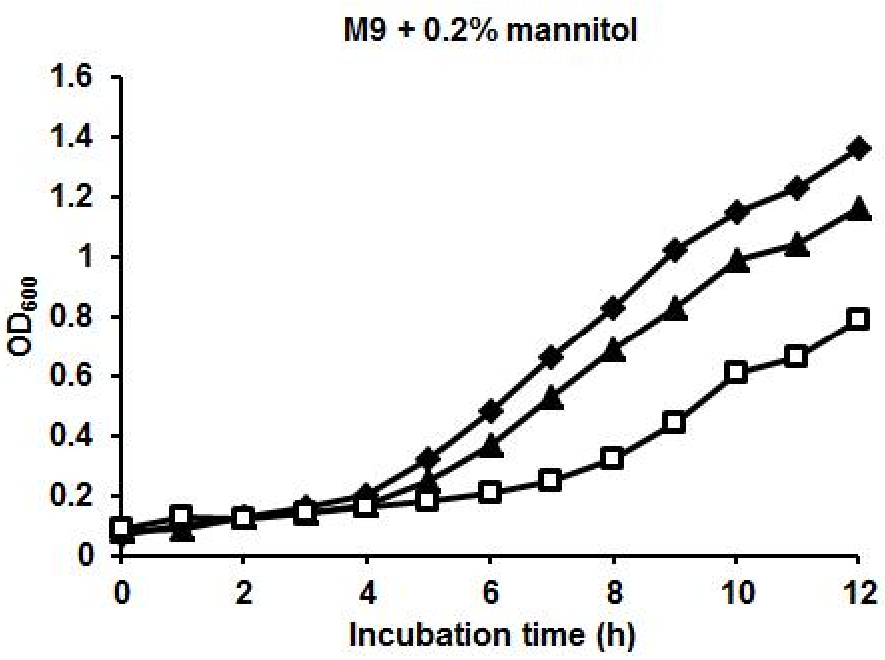

Supplementary Figure 7. Effect of mutant forms of HPr on growth of a *ptsH mtlR* double deletion mutant strain in M9 minimal medium containing mannitol. The growth of the *ptsH mtlR* double deletion mutant strain carrying pACYC-HPr (closed diamonds), pACYC-HPr(K27E) (closed triangles) or pACYC-HPr(L47A/F48A) (open squares) was monitored in M9 minimal medium containing 0.2% mannitol as the sole carbon source at 37 °C.

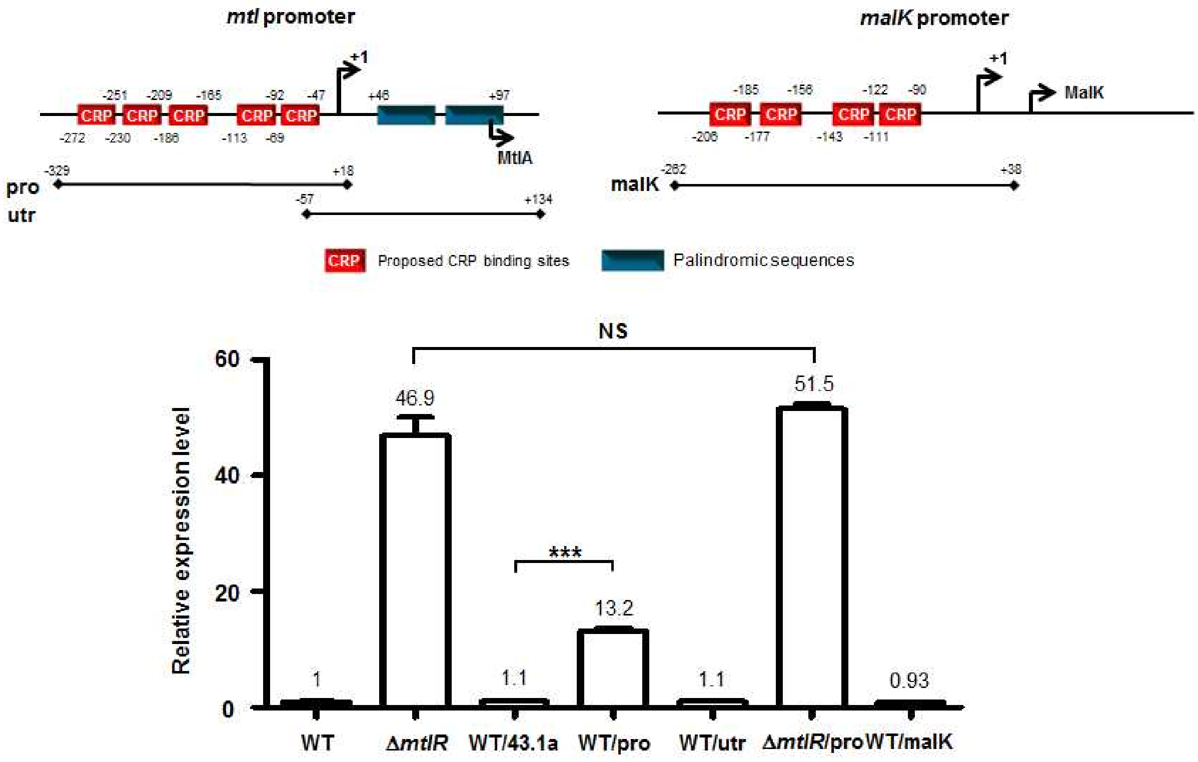

Supplementary Figure 8. Evidence for the specific interaction of MtlR with the promoter region of the *mtl* operon. WT *E. coli* MG1655 or an *mtlR* deletion mutant was transformed with pET43.1a (control plasmid), pET-pro bearing the promoter region of the *mtl* operon (pro), pET-utr carrying a DNA fragment covering the 5'-untranslated region of *mtlA* (utr), or pET-malK carrying the promoter region of the *malK* operon (malK), as indicated, and cultivated in LB medium. The relative transcript level of *mtlA* was determined in cells from early exponential phase by qRT-PCR. Representative data (mean ± SD) from three independent experiments (n=3 each) are shown, and statistical significance was determined by Student's *t*-test (***, $P < 0.001$; NS, not significant). The arrow marked with +1 on DNA represents the transcription start site (TSS) and the arrows marked with MtlA or MalK represent the translation start site of MtlA or MalK. The numbers marked on DNA correspond to the positions relative to the TSS. CRP binding sites were proposed in a previous report [12,13].

Supplementary References

1 Blattner, F. R. *et al.* The complete genome sequence of *Escherichia coli* K-12. *Science* **277**, 1453-1462 (1997).

2 Park, Y. H., Lee, C. R., Choe, M. & Seok, Y. J. HPr antagonizes the anti-s70 activity of Rsd in *Escherichia coli*. *Proc Natl Acad Sci U S A* **110**, 21142-21147, doi:10.1073/pnas.1316629111 (2013).

3 LaVallie, E. R. *et al.* A thioredoxin gene fusion expression system that circumvents inclusion body formation in the *E. coli* cytoplasm. *Biotechnology (N Y)* **11**, 187-193 (1993).

4 Nosworthy, N. J. *et al.* Phosphorylation destabilizes the amino-terminal domain of enzyme I of the *Escherichia coli* phosphoenolpyruvate:sugar phosphotransferase system. *Biochemistry* **37**, 6718-6726, doi:10.1021/bi980126x (1998).

5 Reddy, P., Peterkofsky, A. & McKenney, K. Hyperexpression and purification of *Escherichia coli* adenylate cyclase using a vector designed for expression of lethal gene products. *Nucleic Acids Res* **17**, 10473-10488 (1989).

6 Garrett, D. S., Seok, Y. J., Peterkofsky, A., Clore, G. M. & Gronenborn, A. M. Identification by NMR of the binding surface for the histidine-containing phosphocarrier protein HPr on the N-terminal domain of enzyme I of the *Escherichia coli* phosphotransferase system. *Biochemistry* **36**, 4393-4398, doi:10.1021/bi970221q (1997).

7 Park, Y. H., Um, S. H., Song, S., Seok, Y. J. & Ha, N. C. Structural basis for the sequestration of the anti-s^{70} factor Rsd from $sigma^{70}$ by the histidine-containing phosphocarrier protein HPr. *Acta Crystallogr D Biol Crystallogr* **71**, 1998-2008, doi:10.1107/S1399004715013759 (2015).

8 Chang, A. C. & Cohen, S. N. Construction and characterization of amplifiable multicopy DNA cloning vehicles derived from the P15A cryptic miniplasmid. *J Bacteriol* **134**, 1141-1156 (1978).

9 Bolivar, F., Rodriguez, R. L., Betlach, M. C. & Boyer, H. W. Construction and characterization of new cloning vehicles. I. Ampicillin-resistant derivatives of the plasmid pMB9. *Gene* **2**, 75-93 (1977).

10 Datsenko, K. A. & Wanner, B. L. One-step inactivation of chromosomal genes in *Escherichia coli* K-12 using PCR products. *Proc Natl Acad Sci U S A* **97**, 6640-6645, doi:10.1073/pnas.120163297 (2000).

11 Kim, H. M., Park, Y. H., Yoon, C. K. & Seok, Y. J. Histidine phosphocarrier protein regulates pyruvate kinase A activity in response to glucose in *Vibrio vulnificus*. *Mol Microbiol* **96**, 293-305, doi:10.1111/mmi.12936 (2015).

12 Ramseier, T. M. & Saier, M. H., Jr. cAMP-cAMP receptor protein complex: five binding sites in the control region of the *Escherichia coli* mannitol operon. *Microbiology* **141 (Pt 8)**, 1901-1907, doi:10.1099/13500872-141-8-1901 (1995).

13 Vidal-Ingigliardi, D. & Raibaud, O. Three adjacent binding sites for cAMP receptor protein are involved in the activation of the divergent malEp-malKp promoters. *Proc Natl Acad Sci U S A* **88**, 229-233 (1991).
